# Atrial septum fat deposition and atrial anatomy assessed by cardiac magnetic resonance: relationship to atrial electrophysiology

**DOI:** 10.1186/1532-429X-14-S1-P209

**Published:** 2012-02-01

**Authors:** Patricia B Rizzi, Nathan Mewton, Larisa G Tereshchencko, David G Strauss, Chia-Ying Liu, Richard L Verrier, Francis E Marchlinski, Christopher Cox, Bruce D Nearing, Peter M Spooner, Peter Kellman, Joao A Lima

**Affiliations:** 1 Cardiology, Jonhs Hopkins University, Baltimore, MD, USA; 2 Office of Science and Engineering Laboratories, Center for Devices and Radiological Health,, U.S. Food and Drug Administration, Silver Spring, MD, USA; 3 Beth Israel Deaconess Medical Center, Harvard Medical School, Boston, MA, USA; 4 Division of Cardiology, Department of Medicine, Hospital of the University of Pennsylvania, Philadelphia, PA, USA; 5 Johns Hopkins Bloomberg School of Public Health, Baltimore, MD, USA; 6 Medical Imaging Section Laboratory of Cardiac Energetics, National Heart Lung and Blood Institute, Bethesda, MD, USA

## Summary

To assess the prevalence of fat deposition in the atrial septum with and its relationship with 12-lead electrocardiogram (ECG) atrial parameters (PR interval, P wave duration) and the presence of atrial fibrillation.

## Background

Recent advances in cardiac magnetic resonance (CMR) allow the assessment of even small amounts of fat in the interatrial septum. Its presence has been associated with obesity and supraventricular arrhythmias1,2.

## Methods

44 consecutive patients with normal left ventricular ejection fraction (LVEF> 35%), age<70 years old, were enrolled in a research study exploring the underlying myocardial substrate of ECG findings.

The presence of interatrial fat was assessed with a complete CMR study including Dark-blood DIR-prepared Fat-Water-separated sequence3 in the horizontal longitudinal axis (4 chamber view). The left, right atria areas and the area of fat in the interatrial septum were measured.

All participants underwent a 12-lead ECG recording at rest as well as a clinical interview on the same day as the CMR. The P wave duration, PR interval and the atrial fibrillation (AF) status were recorded.

## Results

The mean age of this population was 59±9 years old and 34 (77%) were male, with a mean BMI of 29.2±4.5 kg/m2. 6 (14%) patients were in AF upon enrollment and 7(16%) had a history of paroxysmal AF.

The mean left atrium area was of 22.5±6.9 cm2 and the mean right atrial area of 21.6±7.7cm2. Interatrial fat was present in 15(66%) of the patients. There was no significant difference in the prevalence of interatrial fat between AF and sinus rhythm patients (p=0.96). There was a non-significant trend towards a smaller fat area in AF patients compared to sinus rhythm (SR) patients (0.13±0.15 cm2 versus 0.45± 0.7 cm2; p=0.3). The right and left atria were significantly enlarged in AF patients compared to SR patients (p=0.0015 and p<0.0001 respectively).

In SR patients, PR interval and P wave duration were positively and significantly associated with right atrial area (r=0.55; p<0.0001 and r=0.52; p=0.001, respectively), but showed no significant correlation with the left atrial area (r=0.27; p=0.10 and r=0.26; p=0.11, respectively).

## Conclusions

The presence of interatrial fat deposition is a common finding in a normal LVEF population and does not appear to be associated with the AF status. RA but not LA area is associated with increase in P wave and PR durations. Further studies in larger group of patients are warranted to further assess the electrophysiologic significance of interatrial fat deposition.

## Funding

Dr. Mewton was partly supported by a post-doctoral research grant from the French Federation of Cardiology.

This project was also part of a research effort funded by a National Institutes of Health grant no. P20HL101397 to Dr. Lima.

**Figure 1:**
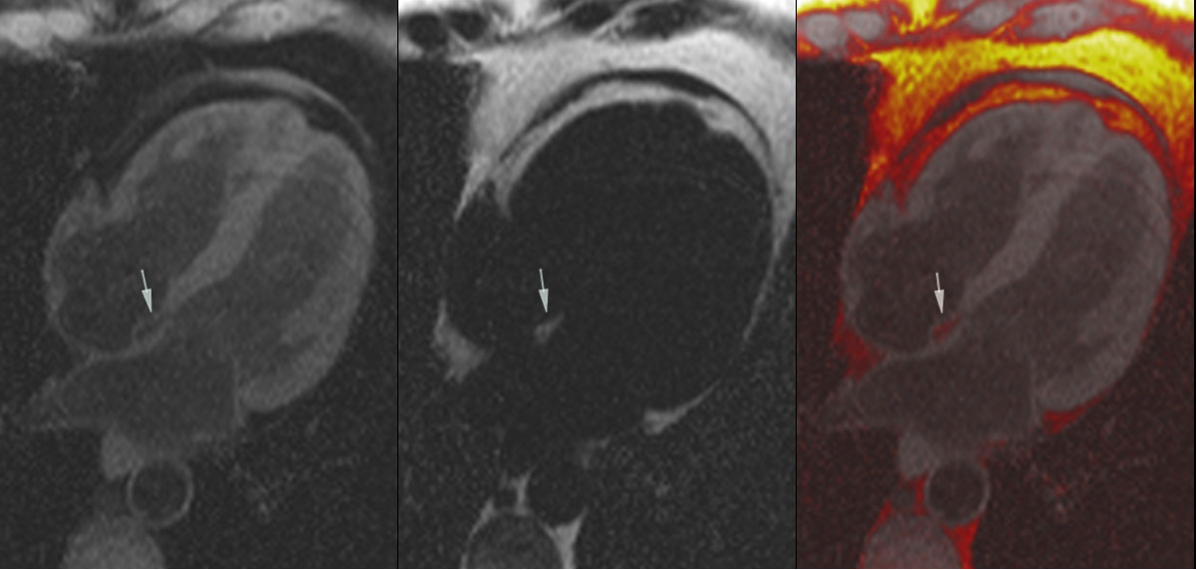
a) Water Imaging, fat deposit in the atrial septum supressed (arrow) b) Fat Imaging, fat deposit in the atrial septum (arrow) c) Fused water-fat imaging, fat deposit in the atrial septum shown in red

